# Standardization in Diverse Populations: Implementation of Evidence-Based Practices in a Safety-Net Setting

**DOI:** 10.3928/24748307-20190107-01

**Published:** 2019-02-05

**Authors:** Roy Cherian, Urmimala Sarkar, Elaine C. Khoong, Sara Ackerman, Gato Gourley, Dean Schillinger

Given the prevalence of medication nonadherence (Sabaté, 2003), clinicians and public health researchers are compelled to design, implement, evaluate, and iterate strategies to improve fidelity to medication regimens. Although barriers to medication adherence range from the individual to the structural, addressing the ambiguity, technicality, and variability of traditional instructions are among the most actionable barriers to comprehension and adherence.

In November 2015, the governing body of Zuckerberg San Francisco General Hospital (ZSFG) decided to implement the Universal Medication Schedule (UMS), evidence-based, plain-language medication instructions. The Ambulatory Safety CEnter for iNnovaTion, funded by a National Institutes of Health grant (P30HS023558-01), led this effort. A prior report describes the implementation outcomes at the four sites (Khoong et al., 2018). Despite the UMS's proven ability to promote accurate recall and comprehension among populations at greatest risk of adverse drug events and nonadherence (Bailey, Agarwal, Sleath, Gumusoglu, & Wolf, 2011; Bailey, Sarkar, Chen, Schillinger, & Wolf, 2012) barriers heretofore unknown and unanticipated compromised UMS implementation at ZSFG's outpatient pharmacy. Given the higher prevalence of barriers to medication comprehension in these settings, such as limited health literacy and/or English proficiency (Bailey et al., 2011; Masland, Kang, & Ma, 2011; Tache, Sonnichsen, & Ashcroft, 2011; Zhan et al., 2005), our inability to successfully implement the UMS in the safety-net settings requires further focused attention. Below we elaborate on tensions described in our short report (Cherian et al., 2018).

## Conflicting Demands

The inability to alter the electronic health record to automate prescribing in UMS led the outpatient pharmacy at ZSFG to implement UMS through manual prescription conversion. However, by placing the onus for conversion on front-line pharmacy staff, who were simultaneously facing pressure to reduce turnaround time, leadership placed the value of efficiency in direct conflict with adherence to evidence-based practice. In this context, it is not surprising that efficiency, due to its immediate benefits to pharmacists' workflows, its measurability, and the leadership emphasis, supersedes efficacy, for which effects are more distal and difficult to quantify. Consequently, although the outpatient pharmacy at ZSFG can dispense medications efficiently, the accompanying instructions are of suboptimal efficacy.

The approach taken by the system privileges a narrow definition of efficiency by focusing on the immediate benefit of reduced turnaround time. However, if efficiency is only valuable insofar as it optimizes efficacy, then an initial investment of resources to implement evidence-based practices that could have a greater impact in the long term may be the more efficient approach.

## Professional Culture, Hierarchy, and Power

The professional knowledge, rules, and sensibilities (i.e., culture) that inform pharmacists also impeded UMS implementation. Specifically, pharmacists consider that the UMS neglects the importance of pharmacokinetics in setting administration times that are not equidistant from each other. However, the argument for the UMS over traditional instructions is that it is cognitively, rather than pharmacokinetically, superior. By fixing administration to certain times of the day (e.g., morning, noon, and evening) that are easier to remember than traditional instructions (e.g., every 8 hours), the UMS increases the likelihood that patients will remain adherent and the intended effect of the medication will be apparent, despite a relative lack of pharmacokinetic precision.

In addition, the concern of medico-legal liability among pharmacists and the rigidity of hierarchies in the professional culture of medicine also impeded implementation. Specifically, UMS implementation requires that pharmacists convert BID (*bis in die*) into “take one pill in the morning and take one pill in the evening” rather than translating the Latin verbatim into “take twice a day.” Because UMS implementation required action outside of the traditional scope of work for pharmacists, anxiety in terms of both liability and transgressing authority was heightened. This was especially concerning for medications that are transferred to external pharmacies, resulting in the possibility of patients receiving inconsistent instructions.

Some of these concerns emerge more so from inadequate team-based UMS implementation than the underlying logic of consolidating medications and plain-language instructions. However, in fields where there is a highly regimented professional culture, such as in medicine, professional roles, responsibilities, and capacities can become ossified such that innovations that seek to rearrange hierarchies of power face significant barriers (Callahan, 2010; Conrad & Schneider, 1986). This is important to interrogate because such hierarchies have been implicated in compromising patient safety efforts specifically (Walton, 2006).

## Reproducibility and The Role of Experiential Evidence

Randomized testing of UMS within a diverse, low-income population has demonstrated significant increases in patient comprehension (90%; *p* < .001) of medication instructions when compared to traditional labels, especially among patients with complex medication regimens and limited health literacy (M. S. Wolf, T. C. Davis, L. M. Curtis, A. J. Webb, et al., 2011; M. S. Wolf, L. M. Curtis, K. Waite, et al., 2011). However, pharmacists were skeptical of its applicability in the local context. Pharmacists' reluctance to accept the UMS points to a broader issue when it comes to implementation science, namely the question of whether the findings of randomized controlled trials are relevant and applicable, let alone reproducible. Although the methodological rigor of controlled trials helps ensure that the findings are valid, the way the tested interventions manifest in real-world settings might be very different.

From pharmacists' perspective, patients with complex medication regimens, limited health literacy and English proficiency may successfully administer and consolidate medications in a controlled setting, but in the real-world when other barriers (i.e., child care, erratic schedules) are present, these same patients may not be able to reproduce the same degree of adherence. Despite the evidence in the literature on UMS, (Bailey, Agarwal, et al., 2011; Bailey, Persell, Jacobson, Parker, & Wolf, 2009; Bailey, Sarkar, et al., 2012; M. S. Wolf, T. C. Davis, L. M. Curtis, A. J. Webb, et al., 2011; M. S. Wolf, L. M. Curtis, K. Waite, et al., 2011; M.S. Wolf, T. C. Davis, Curtis L. M., S.C. Bailey, et al., 2016.), pharmacists instead relied on their own understanding of the lived experience of the safety-net population. By rejecting the applicability of the UMS in the local setting, pharmacists place evidence-based medicine at odds with experiential evidence, or phronesis. Just as the evidence-base for the UMS is valid, so too is the lived experience of clinicians in the safety-net. To resolve the tension between the two, active evaluation in the local setting is necessary and ongoing.

## Epistemology and Praxis: Individual Versus Population Health

Pharmacists claim that high levels of vulnerability and variability within the patient population requires personalized, as opposed to standardized, medication schedules. Pharmacists argue that the UMS administration times are structured around a middle-class lifestyle that is not representative of patients in the safety-net who have structural barriers to care and adherence (Greene, 2004). For reasons ranging from the socioeconomic to the religious, patients might lack the capacity or desire to align their administration with the highly structured format of the UMS.

Although seemingly counterintuitive in the realm of population health, pharmacists' views echo the paradigm of patient-centered care (Anderson, 2002), defined by the Institute of Medicine as “providing care that is respectful of and responsive to individual patient preferences, needs, and values” (Institute of Medicine, 2001, p. 6). For pharmacists, the UMS makes certain assumptions that may not be accurate due to inadequate attention to real-world variation and furthermore do not align with their own experience or that of their patients, who face vulnerabilities including irregular work schedules, housing instability, and inadequate childcare in addition to an array of other factors that make their schedules highly variable.

From the pharmacists' perspective, the UMS contains an implicit assumption that people have a predictable daily routine, and is therefore less responsive to the uncertainties and instabilities that characterize the lives of vulnerable patient populations receiving care in the safety-net. Although the standardized, plain-language of the UMS is itself patient-centered, it operates on the level of the population. By focusing on the level of the individual, pharmacists consider tailored medication schedules to be more substantively patient-centered because to tailor medication schedules, it may be necessary to know how the specific circumstances of individual patients' lives might affect adherence.

However, pharmacists' resistance to the UMS is notable given the fact that there has been little to no resistance to what is arguably a more structured form of dispensing—blister packs, which not only cluster medications but also suggest times for administration in a fashion similar to those of the UMS. Perhaps pharmacists' rejection of the UMS is more of a reflection of the dearth of resources to implement novel interventions than the concept of medication consolidation itself. In other words, resistance to one and not the other might simply be the result of one having associated and established workflows. Before making any conclusions regarding applicability and replicability in the safety-net, greater resources must be put toward establishing the UMS into workflows in ways more than resource-intensive blister packs have been.

## The Role of Standards in Patient-Centered Care

By asserting the incompatibility of standardization in vulnerable populations, pharmacists' position resonates with the sociological literature on standardization, which argues that the push for standardization is an impractical attempt to “render the world equivalent across cultures, time and geography” (Timmermans & Epstein, 2010). The push for standardization that accompanies globalization emerges from a desire to align the beliefs and behaviors of those populations being brought into the realm of international relations with the political and economic interests of the powerful (Higgins & Hallstrom, 2007). Far from neutral, standardization is a value-laden exercise in social regulation and discipline that makes claims of representing an ideal or fundamental truth that is historically and ideologically contingent (Foucault, 1995; Timmermans & Epstein, 2010). To view standardization as a neutral process is to ignore how it is an exercise of power that is not necessarily aligned with the interests or lived experiences of patients themselves. Therefore, implementing standardization in safety-net settings requires extra caution and attention to its limitations as well as its wider social function and implications.

## Conclusion

In closing, we urge readers to take heed not only of the evidence-base of interventions, but to anticipate the potential barriers in real-world settings that span from the individual to the cultural and the structural. In doing so, we may be better primed to anticipate potential barriers to life-saving interventions in ways that allow us to adapt. Recognizing that standardization might be misaligned with the reality of a highly variable and globalizing world compels us to consider that vulnerable patients may require more, not less, attention. The question remains as to how, as providing medical care on the population level requires the rationing of resources.

Although personalized medication schedules would likely benefit anyone receiving them, the greatest benefit lays in redistributing resources to where they are lacking and the prevalence of disease, illness, and suffering is high. Given that the implicit structure in the UMS already corresponds to and accommodates middle and upper-middle class lifestyles, the UMS is likely adequate for wealthier, less vulnerable populations, whereas it may fall short in the precariat class and among racial/ethnic minorities.

However, there is no reason to assume that the UMS is entirely inapplicable in the safety-net either or that vulnerable populations would not benefit from clustering administration times. Nevertheless, these groups might be better served by strategies that are informed based on patients' need, not systems' capacity, and critically address structural vulnerability—equitable, as opposed to equal care (Greene, 2004; Metzl & Hansen, 2014). Critically addressing structural vulnerability, such as limited health literacy, requires community-based research and action that are embedded within and informed both by patients' lives and reliant upon their engagement with the systems that disproportionately care for them. If standards emerge through this interactive process, they will be aligned with both patients' interests and health system capacities in ways that minimize stigmatization and marginalization and work toward realizing health equity.

Therefore, perhaps a better approach may be to apply the UMS in all settings but supplement it with a more tailored regimen when necessary, matching systems' resources with patients' needs and capabilities. Rather than putting the biomedical and population health models in opposition, team-based efforts should integrate the UMS with assessments of food or housing insecurity and inadequate employment or income, integrating medical care with social services. By taking seriously both the evidence behind the UMS as well as the reality of structural factors that may compromise adherence, integrating individual and population-based approaches to health may help us realize equity without placing unrealistic demands on either patients or providers.
